# PNET périphérique à localisation mésentérique

**DOI:** 10.11604/pamj.2014.18.180.972

**Published:** 2014-06-24

**Authors:** Imane Kamaoui, Mustapha Maaroufi, Leila Chbani, Said Ait Laalim, Siham Tizniti

**Affiliations:** 1 Service de radiologie, CHU Hassan II, Fès, Maroc; 2 Service d'anatomo-pathologie, CHU Hassan II, Fès, Maroc; 3 Service de chirurgie viscérale, CHU Hassan II, Fès, Maroc

**Keywords:** PNET périphérique, PNET mésentérique, cancer, peripheral PNET, mesenteric PNET, cancer

## Abstract

Les tumeurs neuroectodermiques primitives (PNET) mésentériques sont exceptionnelles. Elles appartiennent au groupe des tumeurs neuroectodermiques primitives périphériques et sont assimilées histologiquement aux sarcomes d'Ewing. Le diagnostic repose essentiellement sur les résultats immunohistochimiques et cytogénétiques. Nous nous proposons, à travers un cas de PNET à localisation mésentérique de revoir l'ensemble des aspects cliniques, radiologiques, histopathologiques et thérapeutiques de ce groupe de tumeur.

## Introduction

Les tumeurs neuroectodermiques primitives (PNET) mésentériques sont rares. Elles appartiennent au groupe des PNET périphériques dont les localisations les plus fréquentes sont au niveau osseux (le sarcome osseux d'Ewing) et thoracique (la tumeur d'Askin). Ces tumeurs présentent des caractères histo-pathologiques particuliers et restent de mauvais pronostic. Nous rapportons un nouveau cas de PNET mésentérique, métastatique au niveau du foie.

## Patient et observation

**Figure 1:**
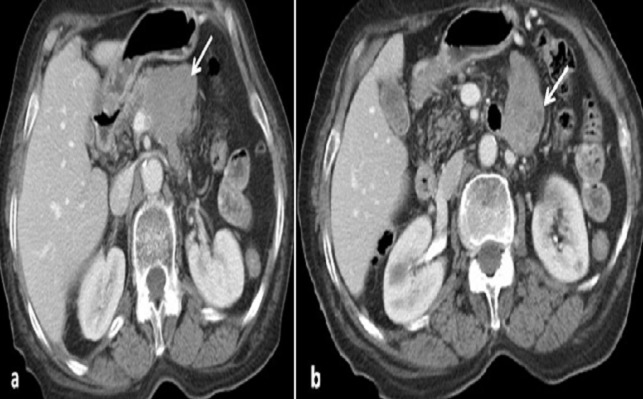
Scanner abdominal en coupe axiale après injection de produit de contraste. Volumineuse masse tumorale de la racine du mésentère, rehaussée de manière hétérogène après contraste (flèche), responsable d'un envahissement du corps du pancréas, du tronc spléno-mésaraique (a) et de l'angle duodéno-jéjunal (b)

**Figure 2:**
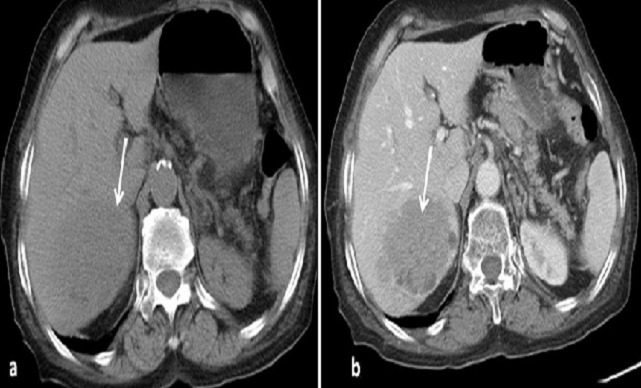
Scanner abdominal en coupe axiale avant (a) puis après injection de produit de contraste (b). Masse hépatique isodense en C-, se rehaussant de manière hétérogène après injection avec zones liquefies

Une femme de 65 ans, sans antécédents particuliers, présente depuis 2 mois des douleurs de l'hypochondre droit (HCD) à type de pesanteur, le tout évoluant dans un contexte d'apyrexie et de conservation de l’état général. L'examen clinique révèle une sensibilité à la palpation abdominale. Le scanner abdomino-pelvien montre une masse de la racine du mésentère, tissulaire, à contours polycycliques, rehaussée de manière hétérogène après injection de produit de contraste délimitant des zones liquéfiées (Figure 1). Cette masse envahit l'angle de TREITZ, le corps pancréatique et le tronc spléno-mésaraique. Le scanner met également en évidence une deuxième masse tissulaire du foie droit, à contours irréguliers, se rehaussant modérément après contraste avec un aspect « pseudokystique ». Cette masse refoule la veine sus hépatique droite sans l'envahir (Figure 2). Le bilan biologique est normal.

**Figure 3:**
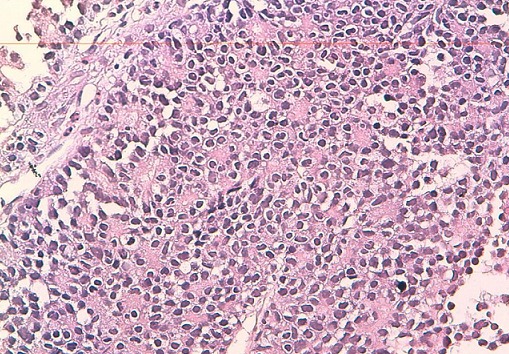
Microscopie (HESX250). Prolifération de cellules rondes avec présence de rosettes

Les masses mésentériques et hépatiques sont biopsiées. Les résultats anatomopathologiques mettent en évidence une prolifération tumorale à cellules rondes exprimant le CD99 (Figure 3). L’étude cytogénétique conclue à une tumeur du groupe PNET/EWING à localisation mésentérique et hépatique. Les tumeurs abdominales sont non résécables. La patiente est adressée en oncologie. Malheureusement, la patiente a refusé le traitement et ne s'est jamais présentée aux séances de chimiothérapie.

## Discussion

Les tumeurs neuroectodermiques primitives (PNET) sont des néoplasmes à cellules rondes dont l'origine présumée est la crête neurale. Elles expriment des critères de différentiation neuronale à des degrés variables et se développent en dehors des systèmes nerveux et sympathique. Les formes les plus connues sont le sarcome d'EWING et la tumeur d'ASKIN. L'atteinte mésentérique est exceptionnelle. Seuls quelques cas isolés ont été rapportés dans la littérature [1–4]. Le diagnostic repose sur l'anatomopathologie. Macroscopiquement, la tumeur est blanchâtre, friable, à contour multi loculé avec des remaniements intra-tumoraux à type de nécrose, d'hémorragie et de kystisation [5]. A l'histologie, il s'agit d'une prolifération à petites cellules rondes monomorphes basophiles se disposant en nappes diffuses. La présence d’élément en rosette ou pseudo-rosettes suggère l'origine neuroectodermique. A l'immunohistochimie, ces tumeurs expriment fortement le CD99 (MIC2). L’étude cytogénétique met en évidence une anomalie chromosomique spécifique, la translocation t (11; 22) (q24; q12).

Le tableau clinique est pauvre, dominé par les signes locorégionaux en rapport avec le syndrome de masse (douleurs, masse palpable). Les complications à type d'occlusion peuvent survenir en cas d'extension locorégionale. L'apparition des signes généraux (altération de l’état général, amaigrissement) traduit un stade évolué de la maladie.

Sur le plan biologique, une perturbation de la numération formule sanguine et du bilan inflammatoire sont notés. Certains auteurs rapportent une élévation du taux sérique de la NSE (Neuron Specific Enolase) et suggèrent que le dosage pré et post opératoire de ce marqueur permet le suivi évolutif des patients [6–9]

L'aspect en imagerie est peu contributif. En scanner, en raison du caractère nécrotico-hémorragique, les tumeurs sont hétérodenses avec zones liquéfiées kystiques. Les remaniements hémorragiques sont hyperdenses spontanément. En IRM, les tumeurs sont isointenses au muscle en T1, hyperintenses hétérogène en T2 avec un rehaussement variable après contraste [10]. Le caractère multiloculaire kystique parcourus par des septa est fréquemment rapporté [1, 11]. En échographie, ces tumeurs présentent un aspect hypoéchogène hétérogène avec des zones kystiques [12]. L'imagerie permet également d'apprécier les mensurations de la tumeur, l'extension locorégionale, la présence de métastases et l’évaluation de la réponse tumorale. Les métastases sont fréquemment retrouvées lors du diagnostic initial comme le cas de notre patiente et sont localisées par ordre décroissant au niveau pulmonaire (50%), os (25%), moelle (20%), foie et cerveau [13].

La prise en charge thérapeutique est multidisciplinaire, associant chirurgie, chimiothérapie et radiothérapie. La chirurgie s'adresse aux formes localisées. La chimiothérapie permet d'optimiser le traitement local et de contrôler la maladie métastatique. La radiothérapie adjuvante permet de diminuer le risque de récidive locorégionale. Le pronostic est réservé avec une survie à 5 ans variable entre 65 à 75% dans les formes localisées et chutant à 25% dans les formes métastatiques [14].

## Conclusion

Les PNET périphériques mésentériques sont rares. L'imagerie est peu spécifique. L'aspect tissulaire hétérogène avec zones kystiques doit faire suggérer le diagnostic. La confirmation est essentiellement anatomo-pathologique.
